# Formation of iron oxide nanoparticles for the photooxidation of water: Alteration of finite size effects from ferrihydrite to hematite

**DOI:** 10.1038/s41598-017-12791-9

**Published:** 2017-10-03

**Authors:** Sebastian P. Schwaminger, Rifki Surya, Simon Filser, Andreas Wimmer, Florian Weigl, Paula Fraga-García, Sonja Berensmeier

**Affiliations:** 1 Bioseparation Engineering Group, Department of Mechanical Engineering, Technical University of Munich, 85748 Garching b, München Germany; 2 Non-Equilibrium Chemical Physics, Department of Physics, Technical University of Munich, 85748 Garching b, München Germany; 3 Division of Analytical Chemistry, Department of Chemistry, Technical University of Munich, 85748 Garching b, München Germany

## Abstract

Iron oxide nanoparticles represent a promising low-cost environmentally-friendly material for multiple applications. Especially hematite (α-Fe_2_O_3_) nanoparticles demonstrate great possibilities in energy storage and photoelectrochemistry. A hydrothermal one-pot synthesis can be used to synthesise hematite nanoparticles. Here, the particle formation, nucleation and growth of iron oxide nanoparticles using a FeCl_3_ precursor over time is monitored. The formation of 6-line ferrihydrite seeds of 2–8 nm which grow with reaction time and form clusters followed by a phase transition to ~15 nm hematite particles can be observed with ex situ X-ray diffraction (XRD), transmission electron microscopy (TEM), Raman and UV/Vis spectroscopy. These particles grow with reaction time leading to 40 nm particles after 6 hours. The changes in plasmon and electron transition patterns, observed upon particle transition and growth lead to the possibility of tuning the photoelectrochemical properties. Catalytic activity of the hematite nanoparticles can be proven with visible light irradiation and the use of silver nitrate as scavenger material. The generation of elementary silver is dependent on the particle size of iron oxide nanoparticles while only slight changes can be observed in the oxygen generation. Low-cost nanoscale hematite, offers a range of future applications for artificial photosynthesis.

## Introduction

Colloidal iron oxide nanoparticles (IONs) demonstrate a great potential for applications ranging from metallurgical processing^1^ and wastewater treatment^2,3^ to medical^4^ and biotechnology^5–8^, energy storage^9–13^ and catalysis^13–15^. A highly investigated research field is the photoelectrolysis of water with hematite due to the global demand for hydrogen with dwindling fossil energy sources. Hematite represents a low-cost photocatalyst for water oxidation as it is the most common iron ore and a semiconductor with a bandgap around 1.9–2.2 eV^16–30^. By the generation of oxygen under light irradiation it can compete with other common photocatalyst systems such as titania^18^. As hematite solely works as photoanode, a coupling to cathode materials such as Pt, Co, Ni, Ir or Ru based catalysts is mandatory to build an electrochemical cell^18,23,31,32^. However, biological materials like phycocyanin proteins, blue green algae or bacteria which can be used conjugated to electrodes in bio-photoelectrochemical cells also represent an interesting possibility for artificial photosynthesis^33^. Furthermore, the biotechnological production of chemicals might be possible with photoanodes as some microorganisms are able to grow and express molecules by electro-fermentation^34^.

Several different bottom-up approaches for generating hematite particles exist aside from the metallurgical exploitation of macroscopic hematite. The higher expenses for syntheses of nanoparticles are justified as the optical properties such as the band gap of IONs can be tuned owing to quantum size effects^35^. Different bottom-up processes from precipitation^36^, sol-gel methods^37^ solvothermal and hydrothermal syntheses^38–40^ to pyrolysis^41^ and combustion^42^ are employed to generate IONs. The shape, size and morphology can be tuned with these methods enabling unique particle properties^36,43,44^. Hydrolysis of FeCl_3_ precursors represents a great possibility to synthesise monodisperse hematite nanoparticles as chloride ions induce aggregation and lead to larger nanoparticles^45^. However, the hydrolysis of iron chloride, the formation of ferrihydrite and the transition to hematite are not completely understood^16,40,46^. Ferrihydrite represents the first stable product of ferric precursor hydrolysis in water^47–49^. The small ferrihydrite seeds nucleate and strongly aggregate to colloidally stable particle clusters depending on pH, ionic strength and ion species^16^. Ferrihydrite also represents a high applicability in wastewater treatment^50,51^. Due to excellent properties for filtering applications with a high specific surface area and reactivity^52^, the precipitation of ferrihydrite is widely investigated^48,53,54^. Ferrihydrite nanoparticles, which act as iron ion storage for plants and microbes, possess a tunable band gap between 1.3 and 2.5 eV and can be used in adsorption of heavy metal ions and metallurgical processing as well as in lithium ion batteries^10,11,50–52,55,56^. The further transition of ferrihydrite to more stable products such as akaganéite, goethite and hematite is dependent on temperature, pH and composition of the solution^16,53,55,57–60^. The transition of ferrihydrite to hematite can occur via dissolution and recrystallisation or topotactic solid state transition which is discussed in literature^46,61,62^. Also many different growth mechanisms for hematite nanoparticles are hypothesised such as aggregation and phase transformation, oriented attachment of hematite particles or precipitation of dissolved ferrihydrite on hematite nuclei^57^.

In this study, we want to focus on the nucleation, phase transition and growth of IONs in order to achieve tunable particle sizes for further applications. Therefore, the phase composition of nanoparticles is investigated after different reaction times of an iron(III)chloride precursor at 98 °C by spectroscopic, microscopic and diffraction techniques with the aim to understanding how particles interact with each other and nucleate at elevated temperatures. Furthermore, the particles are tested towards their properties for the photooxidation of water depending on their size.

## Results

### Characterisation

**Figure 1:**
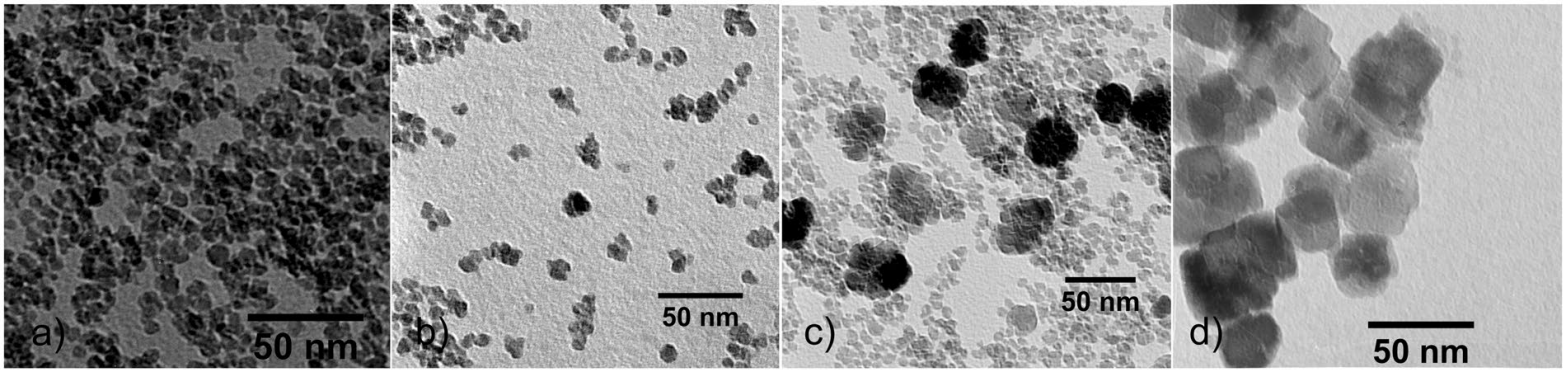
TEM pictures of nanoparticles after (**a**) 10 min, (**b**) 30 min, (**c**) 2 h and (**d**) 6 h of synthesis.

**Figure 2:**
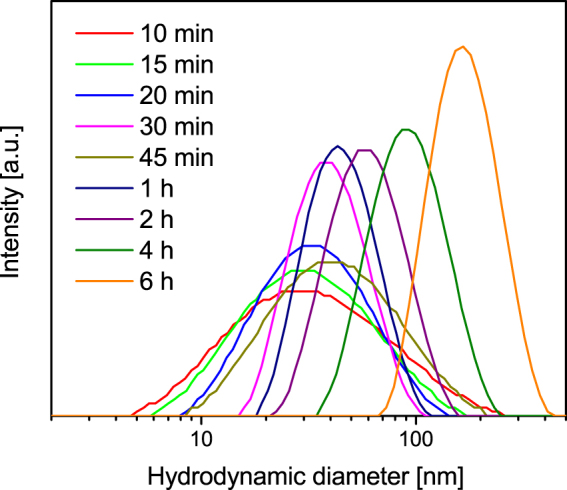
Particle size distributions obtained with DLS measurements of nanoparticles after different synthesis times.

The thermal hydrolysis of iron(III)chloride yields increasing particle size distributions with reaction time as observed by several characterisation methods (Figure S1). This trend for the synthesised nanoparticles is evident from transmission electron microscopy (TEM) pictures shown in Fig. 1 and dynamic light scattering (DLS) analysis, illustrated in Fig. 2. While the TEM analysis shows small spherically to cubically shaped nanoparticles which increase moderately with reaction time and form aggregates, the DLS measurements indicate a significantly larger hydrodynamic diameter for all particles investigated. Especially at long reaction times, the hydrodynamic diameter diverges from the measured TEM diameter due to agglomeration of hematite nanoparticles. An increase in particle size with time as well as an increasing polydispersity can be observed by the TEM particle size distributions (Figure S2). Especially after one hour the diversity of particle sizes is significantly increased. After 2 and 3 hours two size populations develop around 12 and 35 nm. After 4 hours only large sized particles around 35 nm sustain and no small nanoparticles can be observed anymore. The particle size distributions obtained by DLS are quite narrow with polydispersity indices below 0.2. Both methods indicate the coexistence of smaller agglomerated particles and larger particles at elevated reaction times. This offset in particle diameter can be explained with the tendency of the small ferrihydrite nanoparticles to form agglomerates^48^.

**Figure 3:**
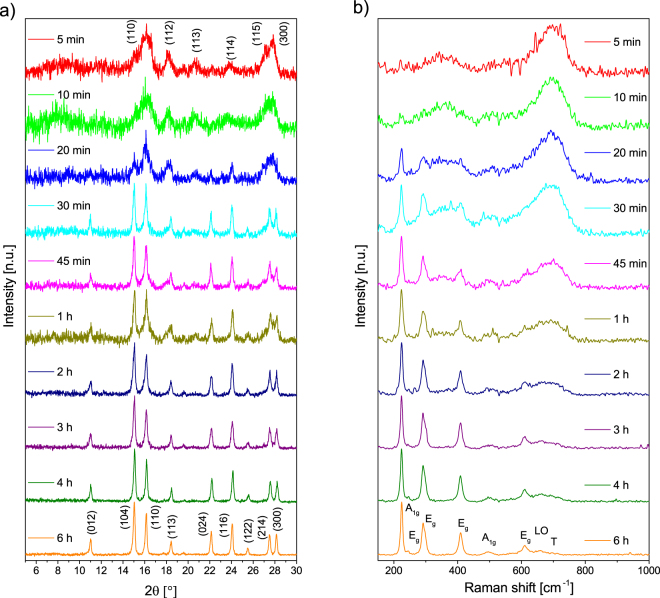
XRD (**a**) and Raman spectra (**b**) of aliquots withdrawn after different reaction times.

Powder XRD yields the crystallinity of the synthesised nanoparticles which can be observed in Fig. 3a. From the full width at half maximum (FWHM) of the reflections, the particle size can be estimated with the Scherrer equation shown in the supplementary information (SI).

In the first 20 minutes, the 6 broad lines (110, 112, 113, 114, 115, 300) shown in Fig. 3a indicating 6-line ferrihydrite are pronounced and decrease in their FWHM with increasing reaction time. After 30 minutes the trigonal crystal structure of hematite can be observed with the two distinct reflections (104 and 110) around 15.0 and 16.1°, respectively. Between the indication of ferrihydrite and hematite, the particle diameter increases significantly according to the FWHM of reflections. Here, the highly crystalline hematite nanoparticles can overlap with the poorly crystalline ferrihydrite and therefore falsify the composition of the material after the respective reaction time. After 30 minutes, the reflections corresponding to hematite are more pronounced and a final diameter of 20 nm can be calculated for the diffractogram after 6 hours. The lattice parameters of hematite nanoparticles are in good agreement with bulk hematite after 30 min reaction time (JCPDS 86-0550). A similar trend is visible in the Raman spectra shown in Fig. 3b. Here, in the 5 and 10 min spectra only Raman spectra of ferrihydrite with broad lines around 360, 510 and 710 nm^−1^ are visible^58^. With increasing reaction time, seven lines corresponding to hematite become more pronounced. Hematite has a corundum structure where the room group D^6^_3d_, five E_g_ (245, 293, 298, 412 and 612 cm^−1^) and two A_1g_ modes (226 and 500 cm^−1^) are active^35^. Furthermore, the disorder related LO mode (660 cm^−1^) and a line corresponding to tetrahedral defects (T band at 690 cm^−1^) are visible in the hematite spectra. These defects also occur in maghemite spectra as well as hydroxide species such as schwertmannite and ferrihydrite which are known to exhibit tetrahedral defects^14,36,61^. Both bands decrease with reaction time as the particles grow larger. However, the T defects are almost completely absent after 6 h of reaction time while the LO mode still shows the same intensity as the E_g_ mode at 610 cm^−1^. With increasing reaction times and according particle sizes, the broadening of all lines corresponding to hematite decreases significantly (Table S1).

For hematite, especially the FWHM of the E_g_ band around 408 cm^−1^ which can be assigned to an oxygen breathing mode is a good indicator for the particle size^61^. We observe a constant decrease in FWHM of this band with reaction time while other modes only show lesser changes (Table S1).

**Figure 4:**
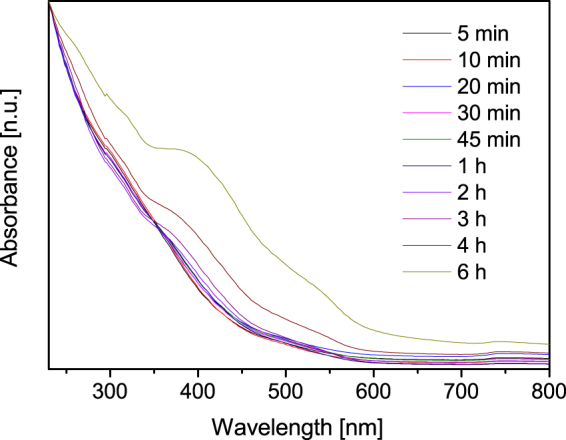
UV/Vis absorbance spectra of aliquots withdrawn after different reaction times.

The optical absorption of nanoscale hematite is based on electron transitions which are therefore decisive for the application of the particles in photooxidation. Hence, the absorption of hematite in the UV and visible region has been thoroughly studied^2,35,43,63^. Fig. 4 shows the change in the absorption spectrum over reaction time. Typical features of hematite become visible for particles larger than 20 nm. While ferrihydrite does not show pronounced absorption lines, hematite has distinct absorption regions around 300 nm, 420 nm and around 550 nm due to different transitions^2^. Different ligand-field transitions depending on the binding state of Fe^3+^ ions as well as ligand-to-metal charge transfer transitions and pair transitions of magnetically coupled electrons of two adjacent Fe^3+^ ions lead to the absorption spectra of the nanoparticles investigated^18,43^. Furthermore, a significant blue shift with decreasing particle sizes can be observed. This behaviour is an indicator for quantum confinement or finite size effects of the synthesised nanoparticles and is in good agreement with literature values for hematite nanoparticles^2,63^. This blue-shift is in agreement with earlier observations of a blue-shifted absorption edge with decreasing sizes for hematite nanoparticles^63,64^. The double excitation process and the corresponding absorption around 535 nm is responsible for the typical red colour of hematite^63^. The absorption of light increases with increasing particle size in this region around 535 nm which can be observed in Fig. 4 with photometry and is also visible to the eye for the hematite nanoparticles in suspension. Furthermore, a significant increase of the region around 400 nm corresponding to ligand field transition is visible in Fig. 4 for increasing synthesis times.

From the absorption spectra, the position of the indirect and direct band gap can be extrapolated as shown in the SI.

**Figure 5:**
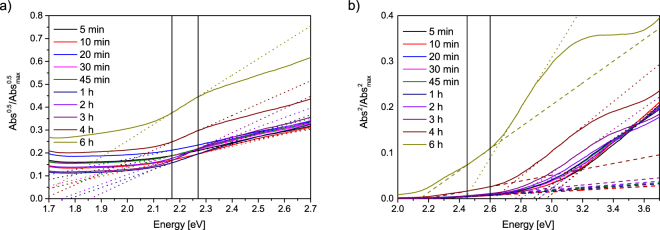
Tauc plots for experimental determination of direct and indirect bandgap (**a**) Square root of normalised absorbance data against photon energy of the aliquots. (**b**) Square of normalised absorbance data against photon energy of the aliquots.

Our extracted values shown in Fig. 5 a for the indirect band gap fit well within the range of literature values for the indirect band gap between 1.38–2.09 eV (Table S2)^2,65,66^. Here, the energy of the band gap increases from the samples taken after 5 minutes to a maximum for the nanoparticles obtained after 3 hour of synthesis. For the 4 and 6 h samples, the band gap energy decreases again. However, the slope and therefore the probability of a transition is steadily increasing from the smallest particles after 5 minutes of synthesis to the largest particles obtained after 6 hours^35^. The energy corresponding to the direct band decreases with particle size from 2.26 to 2.14 eV and are in good agreement with literature values of hematite thin films which range from 1.95 to 2.35 eV^2,35,67^. Here an increase in particle size can be correlated to a lowering of the band gap energy which behaves similar as increasing film thickness of hematite thin films^35^. Moreover, the slope is significantly increasing with increasing particle size by a factor of 10 from the shortest to the longest synthesis time (Table S2).

**Figure 6:**
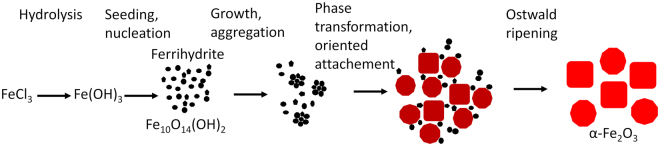
Schematic illustration of hematite synthesis from seeding to growth.

To conclude this characterization section, we want to point out, that we were able to observe the generation of particles in all growth stages for 6 hours. Figure 6 schematically illustrates the synthesis of hematite from the particle seeding, growth and phase transformation to hematite to the final synthesis products. In the first stage around 5–10 minutes, small nuclei form which mainly consist of 6-line ferrihydrite and show a particle size of 2–3 nm. The primary particles grow to 4 nm and nucleate with reaction time to aggregates with hydrodynamic diameters of around 30 nm after 20 min. After 30 minutes the hematite share significantly increases as observed in Raman spectra and XRD results. Here a distinct transition process can be assumed as the hematite particle size derived from the Tauc plot is around 15 nm which is significantly higher than the size corresponding to ferrihydrite and in a similar range to the hydrodynamic diameter of ferrihydrite aggregates. However, still ferrihydrite particles can be observed in Raman spectra as well as XRD after 45 minutes to 1 hour. With increasing reaction times, hematite nanoparticles grow while the amount of ferrihydrite nanoparticles diminishes. We suppose oriented attachment growth where through ferrihydrite adhesion on the larger hematite particles followed by a solid phase transition to hematite by reorientation of iron ions in the crystal lattice. This is also discussed by many groups and is emphasised by the TEM image of particles after 2 hours of synthesis^16,46,57,59^. We cannot completely exclude a dissolution and recrystallisation of ferrihydrite nanoparticles as a parallel mechanism, but the moderately decreasing FWHM of Raman lines and the slow increase in Scherrer diameter rather agrees with oriented attachment growth^57^. With longer reaction times after 3 hours, the particle diameter increases further with reaction time while the surface and tetrahedral defects (LO and T band) decrease which point to an Ostwald ripening process for the particle growth.

With increasing reaction time, the absorption spectrum of hematite also significantly changes leading to more pronounced absorbance lines which shift to higher wave lengths. This behaviour is accompanied by a shift in band gaps of the nanoparticles as well which is in good agreement with literature^35^. A shift of the indirect band gap from 2.26 to 2.14 eV and of the indirect band gap from 1.8 to 1.64 eV as shown in Table S1 might allow the tuning of photocatalytic properties without doping.

### Catalytic activity

While the focus of our investigation is the formation of hematite nanoparticles, the application as photocatalyst represents a further interesting feature. In order to test the catalytic activity, particles synthesised for 45 min, 2 h, 4 h and 6 h were irradiated for 15 min with a 402 nm light source in a closed system. The evolving oxygen was monitored by gas chromatography while the silver ions were measured via induced coupled plasma mass spectrometry (ICP-MS) analysis. The recorded amounts of silver ions and O_2_ were used to calculate the catalytic activity as shown in the SI. A trend of increasing oxygen evolution is evident for increasing nanoparticle diameters and the respective lowering of band gap energies. The highest values obtained for the largest particles shown in Table 1 are in the same range as observed for comparable experiments^17^. The O_2_ production rate increases from 106 to 357 µmol g^−1^ h^−1^ for hematite synthesised for 45 min to 4 h. For the nanoparticles synthesised for 6 h the rate decreases to 324 µmol g^−1^ h^−1^. However, an analysis of the oxygen content at ambient conditions was quite difficult due to fluctuations of room climate with time (Figure S3). Interestingly, the yield of nanoscale silver is significantly higher than the produced amount of O_2_. A similar amount is expected as the generation of photon-induced holes which are able to oxidise water is equal to the produced amount of electrons which are able to reduce Ag^+^ ions. For one oxygen molecule, 4 holes are necessary while one electron is needed in order to reduce silver ions^17^. However, the behaviour for the production is similar as can be seen in Table 1. The rate for silver production, as calculated in the SI, increases from 10 mmol g^−1^ h^−1^ to 44 mmol g^−1^ h^−1^ with increasing particle size from 11 nm (45 min) to 36 nm (4 h). For the particles with sizes around 42 nm synthesised for 6 h the production rate decreases again. The size of produced silver nanoparticles was monitored by single particle (sp)-ICP-MS as described in the SI. The sizes of the produced nanoparticles vary with the different hematite species. While nanoparticles synthesised for 45 min led to silver nanoparticles of sizes around 20 nm, the other photocatalysts led to significantly larger silver particles around 40 nm (Figure S4). Hence, the production rate can benefit from quantum confinement effects. However, the discrepancy between water oxidation and reduction of silver ions means that an optimisation of the system should be possible. A highly likely possibility to explain the discrepancy in oxygen and silver production rates is the subsequent or simultaneous oxidation of silver nanoparticles. Silver nanoparticles are known to form an oxide layer for light irradiations with wavelengths below 495 nm^68^. Grillet *et al*. have demonstrated a photooxidation process of silver nanoparticles around 40 nm under light irradiation^68^. As oxygen is produced by the photoelectrochemical water splitting promoted by hematite nanoparticles, a high local oxygen content and light irradiation can lead to an oxide layer around silver nanoparticles. While it is very difficult to determine an oxide layer around the silver nanoparticles surrounded by smaller iron oxide nanoparticles with TEM (Figure S5), post-catalytic UV-Vis spectroscopy might indicate the formation of an oxide layer (Figure S6). Here, additionally to the absorption lines of hematite, the plasmon peak of silver around 450 nm might be visible. Furthermore, the broadening and a slight redshift of this peak indicates the presence of silver oxide^68^. However, this plasmon peak partially overlaps with the double excitation line of hematite which hinders a precise deconvolution^2,63^. Moreover, Raman spectroscopy emphasises the formation of a silver oxide species after light irradiation (Figure S7). Aside from oxide species, AgNO_3_, which has been used as scavenger material for the catalytic photooxidation of water is visible around 1050 cm^−1^ ^69,70^. The iron oxide species do not significantly change with light irradiation as the A_1g_ mode around 226 cm^−1^ as well as the prominent E_g_ modes around 293 and 412 cm^−1^ are similar to as-synthesised nanoparticles prior to catalytic experiments^35^. The silver oxide layer which is formed around the silver nanoparticles can be observed at around 800 cm^−1^ and at around 600, 350 as well as 200 cm^−1^ where the signals overlap with iron oxide vibrations (Figure S7)^70–73^. Therefore, we assume the formation of an oxide layer around the silver nanoparticles with light irradiation which converts freshly produced oxygen from the photocatalytic activity of nanoscale iron oxide.

**Table 1:** Overview of the nanoparticle sizes, their bandgaps and the catalytic activity. The standard deviation is derived from multiple measurements for TEM and catalytic activity or from the Gaussian fits of the measured distributions for XRD, DLS and ICP-MS.

Time [h]	Size (TEM) [nm]	Size (XRD) [nm]	Size (DLS) [nm]	Bandgap (d) [eV]	Bandgap (id) [eV]	Rate (O2) [µmol g^−1^ h^−1^]	Rate (Silver) [mmolg^−1^ h^−1^]	Size (Silver NP) [nm]
0.75	11 ± 4	13 ± 1	39 ± 4	2.22	1.56	106 ± 58	12 ± 0.1	17 ± 1
2	19 ± 12	15 ± 2	42 ± 4	2.23	1.75	182 ± 12	14 ± 0.1	41 ± 3
4	36 ± 6	19 ± 1	86 ± 20	2.19	1.68	357 ± 18	44 ± 0.2	41 ± 2
6	42 ± 9	19 ± 2	184 ± 14	2.14	1.65	326 ± 45	36 ± 0.5	50 ± 2

## Conclusions

Hydrothermal synthesis of hematite nanoparticles represents a cost-effective possibility to produce photoanodes for artificial photosynthesis.

In this study, we synthesised hematite nanoparticles by a hydrothermal route and observed increasing particle sizes with reaction time. The complementary analysis with TEM, XRD, DLS and Raman spectroscopy during various reaction stages leads to a deeper understanding of the hematite nanoparticles’ formation process. The initial ferrihydrite seeding is followed by aggregation, phase transition to hematite and further oriented attachment growth. The nanoparticles demonstrated quantum confinement and thus a shift in their absorption and band gaps with changing particle diameter. Moreover, the particles demonstrated photocatalytic properties which are influenced by the quantum confinement depending on particle size. The α-Fe_2_O_3_ nanoparticles are able to oxidise water to O_2_ with visible light irradiation in the presence of silver nitrate as scavenger (sacrificial agent) material. We observed a catalytic activity of up to 44 mmol h^−1^ g^−1^ Ag^+^ ions to elementary silver nanoparticles. However, the amount of produced O_2_ was limited to 367 µmol h^−1^ g^−1^. While charge transport might still remain a major problem for the use of hematite in photoanodes, the possibility to tune the band gap without metal ion doping is an important step for the development of artificial photosynthesis. Especially the use of nanoparticles instead of macroscopic electrodes could represent many new possibilities for a multitude of applications. Furthermore, this represents a simple process to produce silver and silver oxide nanoparticles.

## Methods

All reagents applied are commercially available and were used as received from the manufacturer without further purification.

### Synthesis

The co-precipitation of iron oxide nanoparticles was performed in a stirred tank reactor with deionised water and ferric chloride hexahydrate (FeCl3∙6H2O) (Sigma-Aldrich, Germany). Iron oxide nanoparticles were obtained by hydrolysis of 950 mL water where 50 mL of a ferric chloride (25 mmol) solution were added dropwise. The solution was held at 98 °C for 6 h under vigorous stirring. After 5 min, 10 min, 15 min, 20 min, 30 min, 45 min, 1 h, 2 h, 3 h, 4 h and 6 h aliquots of 50 mL were withdrawn and cooled with ice. The suspensions were dialysed until the conductivity dropped below 200 µS cm^−1^. A colloidal sample was stored for transmission electron microscopy (TEM) and UV/Vis spectroscopy while the rest of the precipitates were lyophilised with an ALPHA 1-2LDplus (Martin Christ Gefriertrocknungsanlagen GmbH, Germany).

### X-ray diffraction (XRD)

Crystal structure and phase purity of the lyophilised samples were examined with powder XRD. The measurements were performed with a Stadi-P diffractometer (STOE & Cie GmbH, Germany), equipped with a molybdenum source (Ge (111) monochromator, Kα1 radiation (λ = 0.7093 Å)) and a Mythen 1 K detector (DECTRIS Ltd., Switzerland) in transmission geometry. Data was collected in the range from 2° to 50° (2ϴ).

### Raman spectroscopy

Raman spectra were recorded by a SENTERRA spectrometer (Bruker Optics GmbH, Ettlingen, Germany) equipped with a 488 nm laser. Low laser powers (1 mW) were chosen in order to prevent oxidation of the samples. A baseline correction was accomplished with the software OPUS 7.2 for spectra measured by Raman spectroscopy using the concave rubber band method.

### Transmission electron microscopy (TEM)

The particle dimensions were assessed by TEM using a JEM 100-CX (JEOL GmbH, Germany). For the TEM measurements the colloidal samples were diluted in degassed and deionised water, treated with ultrasound to disperse any agglomerates and precipitated on carbon coated copper grids (Quantifoil Micro Tools GmbH, Germany). The pictures were manually processed in ImageJ. A number of 50 particles per sample were measured in random order.

### Dynamic lights scattering (DLS)

The hydrodynamic diameter of particles and particle agglomerates was studied by a DLS method with a Delsa Nano C Particle Analyzer (Beckman Coulter GmbH, Germany). Each aliquot was analysed as-synthesised with a 658 nm laser under a 165° scattering angle over 100 single measurements.

### Ultraviolet/visible light (UV/Vis) spectroscopy

UV/Vis spectroscopy measured in triplicates was conducted in microtiter plates of 200 mL particle suspensions in a photospectrometer (Tecan GmbH, Germany). The suspensions were scanned in the region from 230 to 800 nm with 2 nm steps. The spectra were normalised in order to vouchsafe a better comparison of all samples.

### Catalytic activity

Catalytic activity was tested by irradiating 1.2 mL of the ferrrihydrite/hematite samples (45 min, 2 h, 4 h and 6 h) which were incubated with 300 µL of a silver nitrate solution (0.1 M) used as a scavenger for the reaction at pH 6.5 in GC vials. Therefore, the light source Zahner TLS03 with 137 W m^−2^ (87 mm^2^) and a wavelength of 402 nm was used and the samples were irradiated for 15 minutes and turned by 120° every five minutes. The overhead of the solutions was analysed with a GC Shimadzu GC2010 Plus equipped with a barrier ionization discharge (BID 2010 Plus) detector and a Restek Shin Carbon ST 80/100 column.

The supernatant was separated from the particles by centrifugation at 17000 *g*, diluted and analysed with induced coupled plasma mass spectrometry (ICP-MS).

An Agilent 7900 ICP-MS (Agilent Technologies, Santa Clara, CA, USA) was used in the determination of the isotopes^107^Ag and^109^Ag. The instrument was equipped with an Agilent SPS4 autosampler and an Agilent MicroMist nebuliser (U-Series). 500 µL of each sample were diluted in 3% HNO_3_ (2.5 mL HNO_3_) from a concentrated HNO_3_ (65%, p.A., Merck KGaA, Darmstadt, Germany), which was purified using a subboiling device, by the factor of 100 on the same day of measurement in 50 mL vials.

## Electronic supplementary material

Supplementary information
