# Supplementary material for: Formation of iron oxide nanoparticles for the photooxidation of water: Alteration of finite size effects from ferrihydrite to hematite
Source: Sci Rep. 2017 Oct 3;7:12609. doi: 10.1038/s41598-017-12791-9 (PMC5626691; doi:10.1038/s41598-017-12791-9)
Supplement: Supplementary file 1 — Supplementary information [file 41598_2017_12791_MOESM1_ESM.pdf]

SUPPLEMENTARY INFORMATION

# Formation of iron oxide nanoparticles for the photooxidation of water: Alteration of finite size effects from ferrihydrite to hematite

**Sebastian P. Schwaminger[1], Rifki Surya[1], Simon Filser[2], Andreas Wimmer[3], Florian Weigl[3], Paula Fraga-García[1] and Sonja Berensmeier[1*]**

[1]Bioseparation Engineering Group, Department of Mechanical Engineering, Technical University of Munich, 85748 Garching b. München, Germany

[2]Non-Equilibrium Chemical Physics, Department of Physics, Technical University of Munich, 85748 Garching b. München, Germany

[3]Division of Analytical Chemistry, Department of Chemistry, Technical University of Munich, 85748 Garching b. München, Germany

*corresponding author: s.berensmeier@tum.de

## Calculations

### Primary particle diameter

From the Scherrer equation the primary particle diameter can be estimated. Here we use the shape factor B as 0.89, λ of Mo Kα 0.7063 Å, Δθ as FWHM and θ as position of the respective reflection.

$$d = \frac{B \cdot \lambda}{\Delta\theta \cdot \cos\frac{\theta}{2}}$$

### Band gaps

From the absorption spectra, the position of the indirect and direct band gap can be extrapolated. The direct band gap can be calculated with a Tauc plot, shown in the following equation, where the square of the absorbance A is plotted against the difference of photon energy hν and the band gap $E_g$.

$$A \sim \left(h\nu - E_g\right)^{\frac{1}{2}}$$

Accordingly, the indirect band gap of hematite nanoparticles can be estimated with another Tauc plot as the square root of the absorbance here is directly proportional to the difference of photon energy and the band gap.

$$A \sim \left(h\nu - E_g\right)^2$$

## Catalytic activity

From the peak ratio between area increments (A) of $O_2$ (2.8 min) and $N_2$ (3.2 min) signals obtained by the BID, the oxygen content in the headspace ($x_{O2}$) is calculated.

$$x_{O_2} = \frac{A(O_2)}{A(N_2)} \cdot \left(0.99 - \frac{A(O_2)}{A(N_2)}\right)$$

For the oxygen evolution rate (OER), the difference between the sample's oxygen content and the oxygen content of a reference is multiplied with the headspace volume V and divided by the molar volume at standard conditions ($V_m$), by the mass of used catalyst (m) and by the irradiation time (t).

$$OER = \frac{\left(x_{O_2}(Sample) - x_{O_2}(Ref)\right) \cdot V}{V_m \cdot m \cdot t}$$

For the production rate of silver nanoparticles, the amount of ionic silver is measured as supernatant concentration ($c_s$) by ICP-MS. The difference between the original amount of provided $AgNO_3$ ($c_0$) and the obtained supernatant concentration is used as measure for the produced silver nanoparticles as no adsorption of silver ions was observed with a reference sample. Thus the production rate $r_{Ag}$ can be expressed as quotient of the amount of silver nanoparticles and the product of the mass of hematite catalyst and irradiation time.

$$r_{Ag} = \frac{c_0 - c_s}{m \cdot t}$$

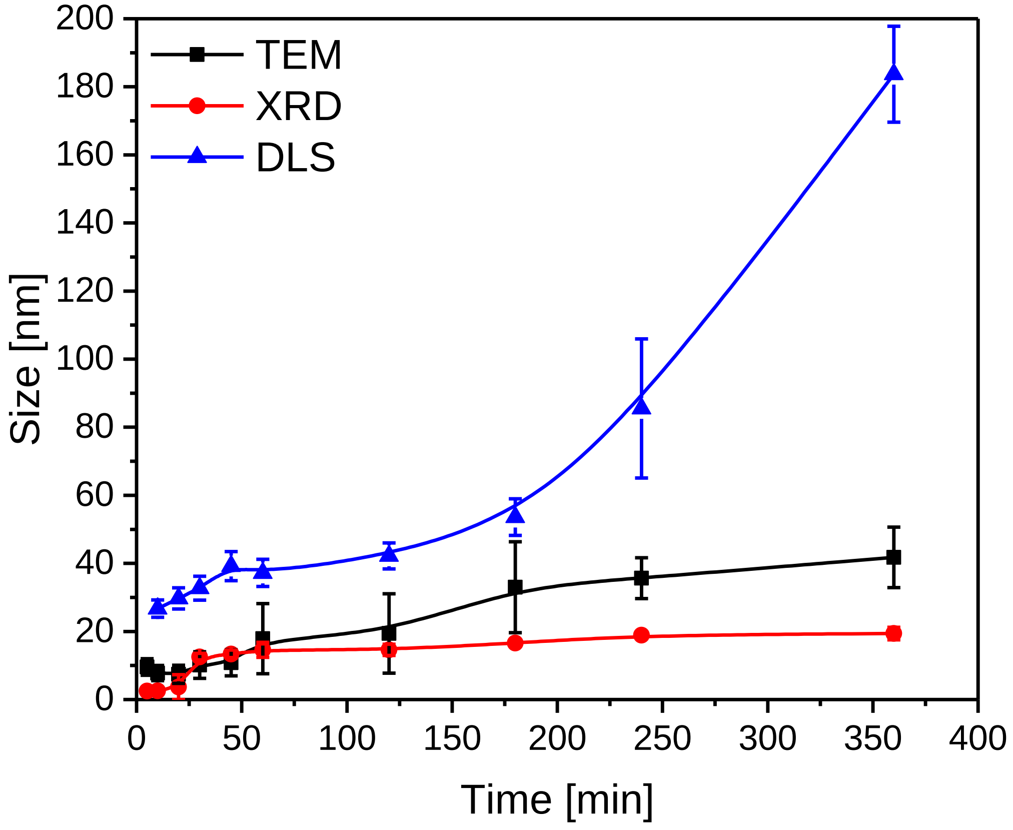


**Figure S1 Size development of nanoparticles with synthesis time as observed by TEM, XRD and DLS.**

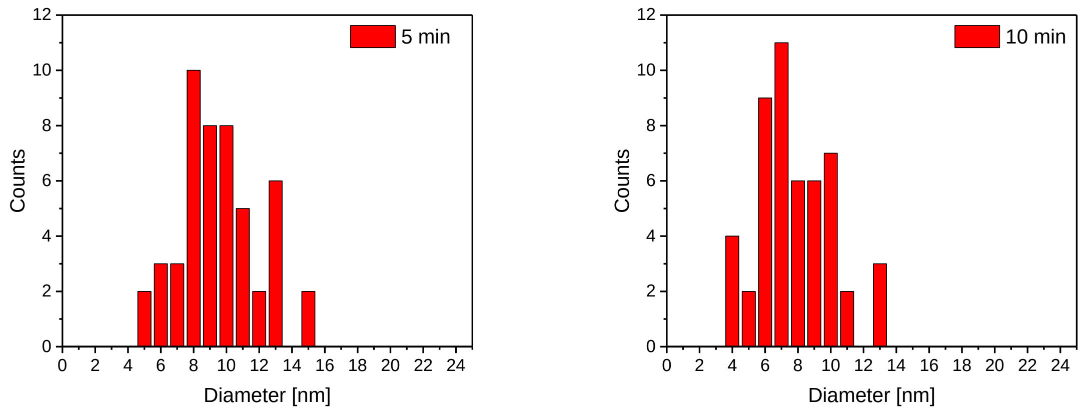

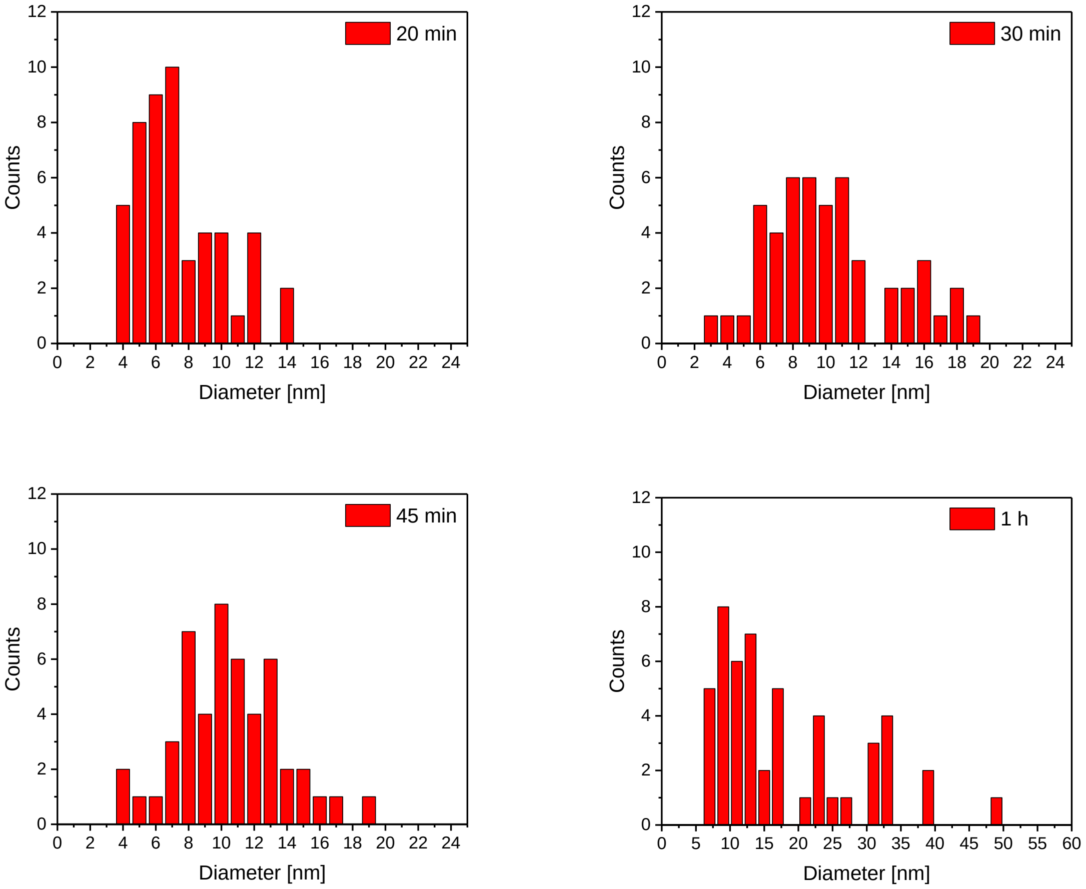
20 min
30 min
45 min
1 h
Counts
Diameter [nm]

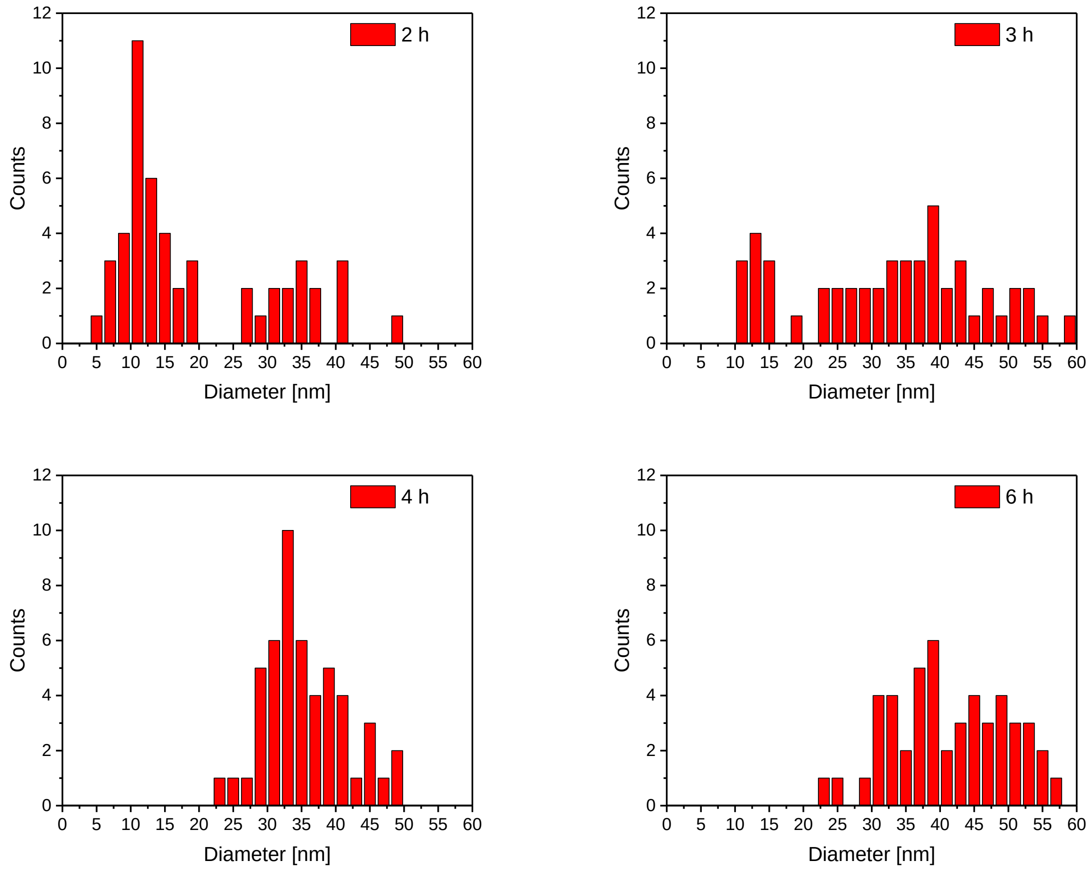


**Figure S2 Size distributions of nanoparticles from different synthesis time as counted from TEM images (50 counts).**

**Table S1 Positions and FWHM of characteristic hematite bands for different synthesis times**

| | A1g | | Eg | | Eg | | LO | | T | |
|---|---|---|---|---|---|---|---|---|---|---|
| Time [min] | Position [$cm^{-1}$] | FWHM [$cm^{-1}$] | Position [$cm^{-1}$] | FWHM [$cm^{-1}$] | Position [$cm^{-1}$] | Area | Position [$cm^{-1}$] | Area [$cm^{-1}$] | Position [$cm^{-1}$] | Area |
| 30 | 223.3 | 11.0 | | | | | | | | |
| 45 | 223.5 | 9.4 | 407.2 | 20.0 | 608.7 | 9.6 | 656.5 | 36.3 | 705.0 | 54.1 |
| 60 | 223.9 | 8.8 | 408.2 | 18.0 | 605.9 | 8.0 | 650.0 | 42.1 | 705.0 | 49.9 |
| 120 | 224.1 | 9.1 | 408.2 | 16.2 | 607.4 | 13.3 | 650.0 | 42.9 | 702.8 | 43.8 |
| 180 | 224.0 | 8.3 | 408.5 | 14.7 | 609.3 | 26.8 | 656.3 | 44.7 | 704.7 | 28.5 |
| 240 | 224.0 | 8.5 | 408.4 | 15.2 | 608.9 | 28.0 | 655.9 | 43.4 | 701.2 | 28.6 |
| 360 | 224.4 | 7.5 | 409.2 | 13.0 | 609.6 | 46.1 | 656.8 | 42.2 | 703.3 | 11.7 |

**Table S2 Bandgap energy positions and slopes in the Tauc plots.**

| | Bandgap (d) I | | Bandgap (d) II | | Bandgap (id) | |
|---|---|---|---|---|---|---|
| Time [min] | Energy [eV] | Slope | Energy [eV] | Slope | Energy [eV] | Slope |
| 5 | 2.22 | 0.02 | 2.96 | 0.27 | 1.32 | 0.22 |
| 10 | 2.25 | 0.02 | 2.96 | 0.28 | 1.53 | 0.26 |
| 20 | 2.18 | 0.02 | 2.91 | 0.25 | 1.24 | 0.23 |
| 30 | 2.22 | 0.02 | 2.91 | 0.26 | 1.49 | 0.28 |
| 45 | 2.22 | 0.02 | 2.88 | 0.24 | 1.56 | 0.31 |
| 60 | 2.26 | 0.02 | 2.87 | 0.25 | 1.78 | 0.40 |
| 120 | 2.23 | 0.03 | 2.80 | 0.22 | 1.75 | 0.42 |
| 180 | 2.25 | 0.03 | 2.76 | 0.24 | 1.86 | 0.53 |
| 240 | 2.19 | 0.06 | 2.62 | 0.27 | 1.68 | 0.50 |
| 360 | 2.14 | 0.24 | 2.44 | 0.55 | 1.65 | 0.72 |

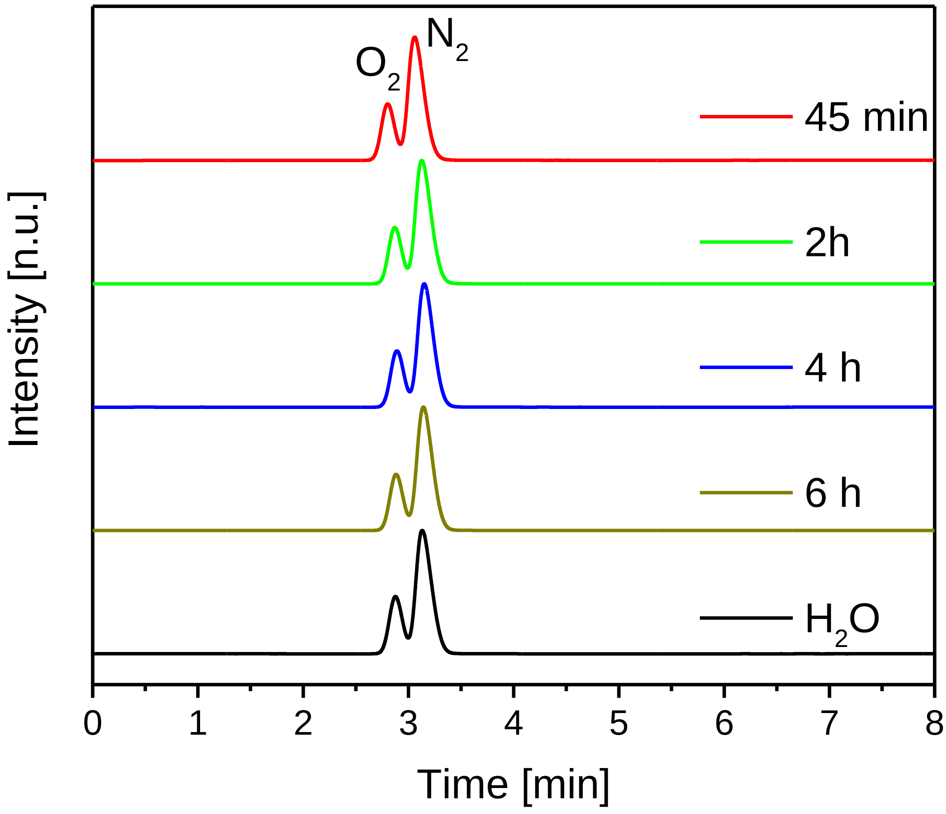


**Figure S3 Gas chromatograms of the headspace of four different hematite and a water reference samples after the irradiation with a light source.**

## Size determination of AgNPs using sp-ICP-MS (single particle induced coupled plasma mass spectrometry)

The irradiated samples were washed several times with VivaSpin™ vials (10000 Da) and diluted prior to ICP-MS analysis. Particle size distributions were determined by sp-ICP-MS measurements using a 7900 ICP-MS in single particle mode equipped with a SPS4 autosampler (Agilent, Santa Clara, CA, USA). In single particle measurements, a time resolved spectra of a target mass reveals information about concentration and particle size distribution of nanoparticles consisting of the target element. Each time a nanoparticle is ionized in the plasma, a sudden increase in the signal intensity is observable. The peak area highly correlates with the particle mass and size. By counting all signal peaks, concentration of the particulate element can be deduced. All samples were diluted with ultra-pure water resulting in final concentrations in the range of 20 to 80 ng $L^{-1}$ Ag. Ultrasonication prior to dilution steps and measurement prevents particle agglomerates. Dwell time was set to 100 µs with an acquisition time of 60 s resulting in 600000 data points each measurement. The pump rate of the peristaltic pump was determined as 0.346 mL $min^{-1}$, and $^{107}Ag$ was selected as target mass. According to a recently published operation procedure,[1] transport efficiency, which describes how many particles of the sample finally reach the detector, was determined using a gold nanoparticle dispersion (NIST reference material 8012, 28 nm, 48 ng $L^{-1}$, Gaithersburg, MD, USA). Since signal intensity is element specific, a second calibration based on an ionic silver standard (1 µg $L^{-1}$ in 1.625% (v/v) $HNO_3$) was used to determine element sensitivity. Particle size distributions were calculated in Agilent's MassHunter software (version C.01.02) equipped with a Single Nanoparticle Application Module.

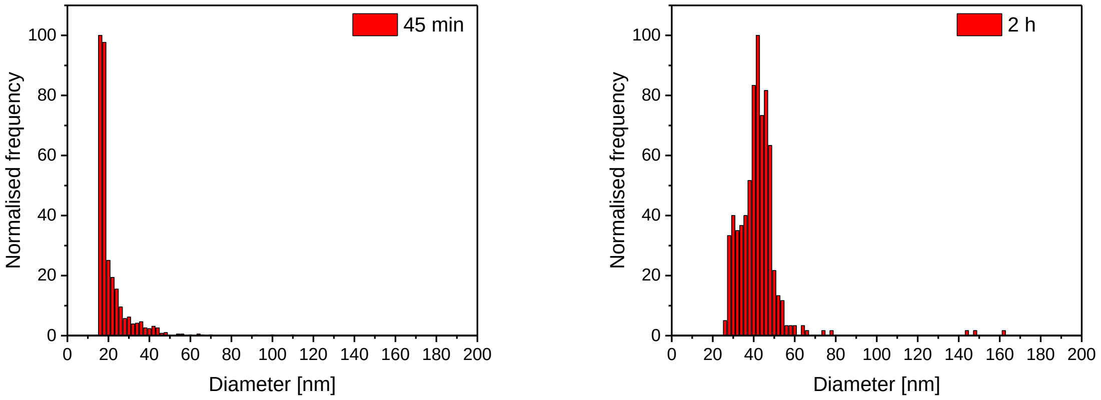

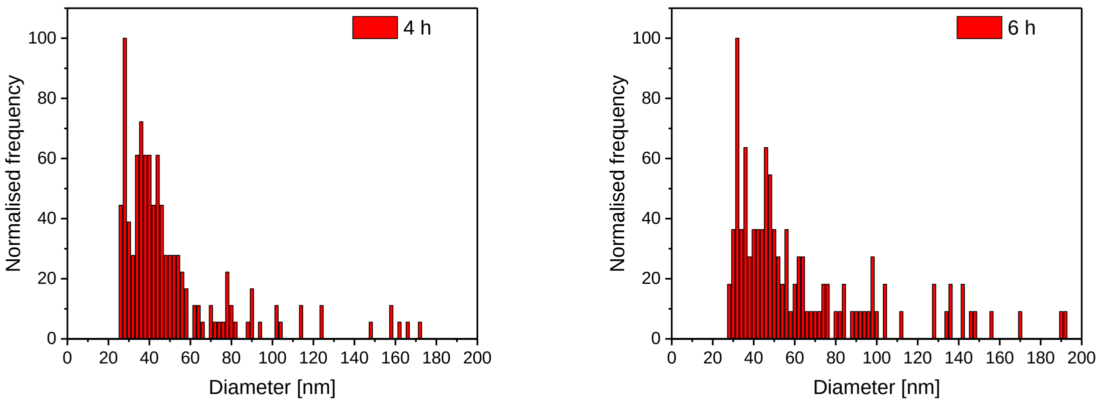


**Figure S4 Size distributions of silver nanoparticles as produced by light irradiation of silver nitrate in the presence of hematite nanoparticles with various synthesis times.**

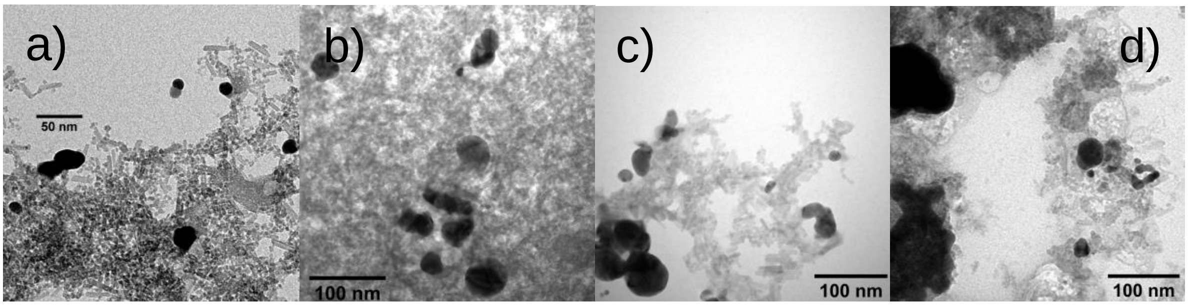


**Figure S5 TEM images of iron oxide nanoparticles and generated silver nanoparticles (black, spherically shaped) after light irradiation for 15 minutes and several washing steps. The particles were synthesized for 45 min (a), 2 h (b), 4 h (c) and 6 h (d).**

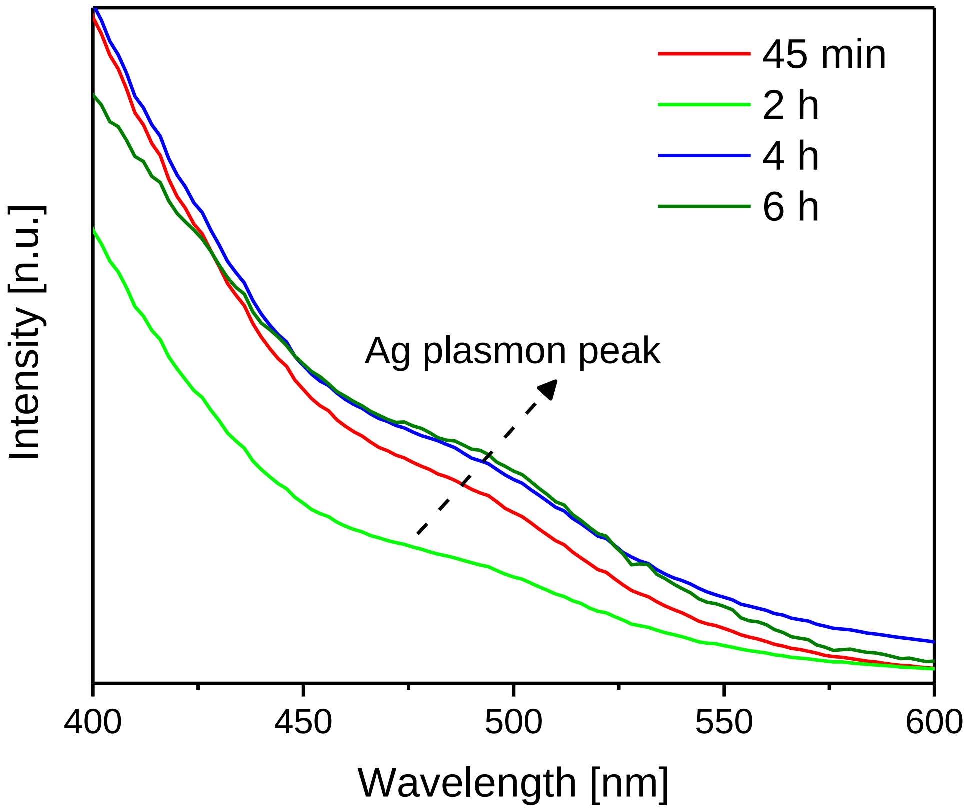


**Figure S6 UV-Vis spectra of iron oxide nanoparticles and generated silver nanoparticles after light irradiation for 15 minutes and several washing steps. The particles were synthesized for 45 min, 2 h, 4 h and 6 h.**

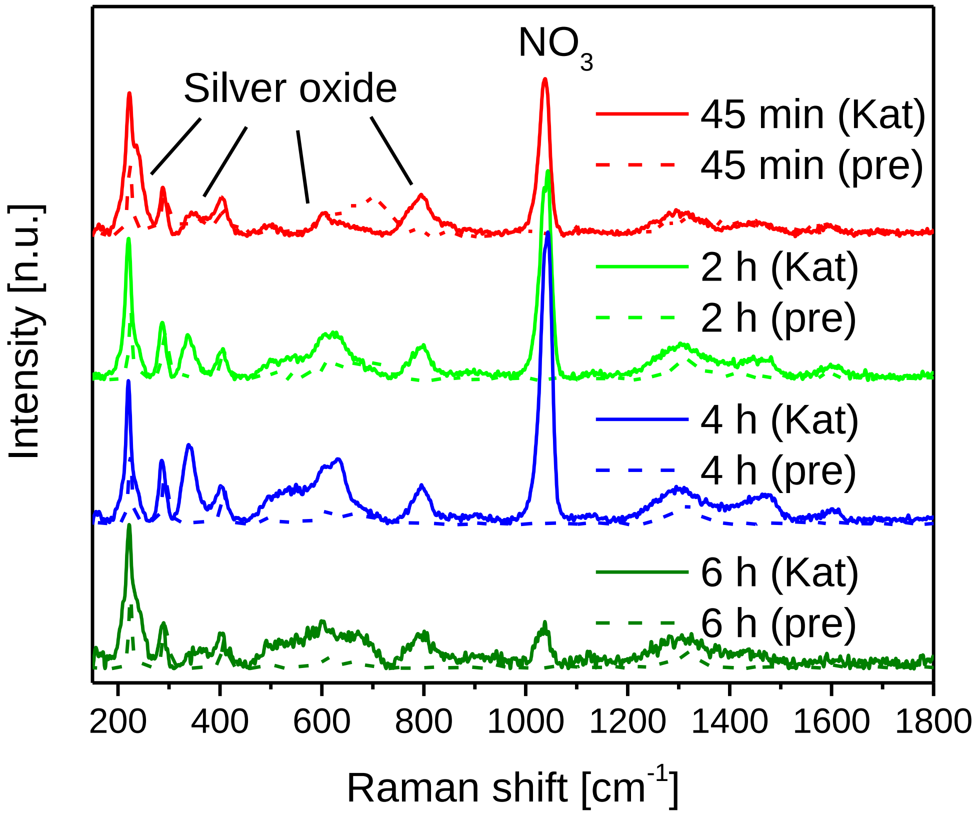


**Figure S7 Raman spectra of iron oxide nanoparticles and generated silver nanoparticles after light irradiation for 15 minutes compared to as synthesized nanoparticles (dashed lines). The particles were synthesized for 45 min, 2 h, 4 h and 6 h.**

## References

1. Peters, R. J. B. *et al.* Development and validation of single particle ICP-MS for sizing and quantitative determination of nano-silver in chicken meat. *Anal. Bioanal. Chem.* **406,** 3875–3885 (2014).
